# Effects of music listening on anaerobic performance and motivation in healthy young adults

**DOI:** 10.3389/fspor.2025.1518359

**Published:** 2025-03-14

**Authors:** Luca Cavaggioni, Damiano Formenti, Ibrahim Ouergui, David Perpetuini, Paolo Castiglioni, Alessandro Berengan, Athos Trecroci, Luca Paolo Ardigò, Giampiero Merati

**Affiliations:** ^1^ Department of Biotechnology and Life Sciences, University of Insubria, Varese, Italy; ^2^ Obesity Unit and Laboratory of Nutrition and Obesity Research, Department of Endocrine and Metabolic Diseases, IRCCS Istituto Auxologico Italiano, Milan, Italy; ^3^ High Institute of Sport and Physical Education of Kef, University of Jendouba, El Kef, Tunisia; ^4^ Research Unit: Sports Science, Health and Movement, University of Jendouba, El Kef, Tunisia; ^5^ Department of Engineering and Geology, University G. D’Annunzio of Chieti-Pescara, Pescara, Italy; ^6^ IRCCS Fondazione Don Carlo Gnocchi Onlus, Milan, Italy; ^7^ Sciences of Preventive and Adapted Physical Activities, University of Insubria, Varese, Italy; ^8^ Department of Biomedical Sciences for Health, Università Degli Studi di Milano, Milan, Italy; ^9^ Department of Teacher Education, NLA University College, Oslo, Norway

**Keywords:** sprint, perceived exertion, motivation, running, music

## Abstract

**Introduction:**

The study aimed to investigate and confirm from a physiological and psychological perspective whether preferred music would influence anaerobic performance during the Running-Based Anaerobic Sprint Test (RAST).

**Methods:**

A total of 18 (men, *n* = 12, women, *n* = 6) sub-élite track-and-field and football athletes (mean age 22.2 ± 2.1 years, mean height 175.3 ± 8.0 cm, mean weight 66.4 ± 10.6 kg, mean BMI 21.5 ± 2.2 kg/m^2^) were voluntarily recruited. The RAST procedure was performed by recording maximum power (Pmax), average power (Pmean), minimum power (Pmin), rating of perceived exertion (RPE), and motivational level (visual analog scale) while listening to preferred or no music through headphones.

**Results:**

Listening to music significantly increased motivation (*p* < 0.001, effect size = 1.31, very large) compared to no music. However, no significant differences were observed in other performance variables between the “with music” and “without music” conditions.

**Conclusions:**

Overall, listening to preferred music during an anaerobic exercise improves motivation as confirmed by previous evidence. This could be helpful for athletes to strive for even higher goals by improving their current performance level.

## 1 Introduction

Playing and listening to music is an enjoyable, widespread, and ancient activity, spanning from early in childhood to older age (1). Today, it is a popular training tool among athletes, valued for its positive effects on affective and emotional states, as well as its ergogenic effects on physical performance (e.g., cardiovascular and respiratory function), perceived exertion, and oxygen consumption (2). In this context, several studies have confirmed the ability of resistance training exercises while listening to music to increase muscular strength performance and the number of repetitions performed during training sessions, and to reduce the perception of fatigue (3, 4). For performance-enhancing purposes, music can be listened to before or during physical exercise. In fact, there is already a growing body of literature on the effects of music listening during the warm-up procedure on both motor performance and psycho-physiological responses to physical effort (5–8). Specifically, pre-task music has been found to enhance psychological responses and alleviate fatigue-related symptoms associated with exercise performance (5). In particular, some authors found that the best result is achieved after listening to preferred music played at 140 bpm and 80 dB (6, 7) and combining music listening with plyometric-based exercise (8). Notably, music listening can be beneficial for both endurance and high-intensity performances, as demonstrated by Patania et al. who showed that the beneficial effects of music are more likely to be observed in endurance exercises (9). As such, a previous study (10) tested participants under four music conditions: fast/loud, fast/quiet, slow/loud, slow/quiet, and without music, during 10-min exercise sessions on a treadmill, revealing that the speed produced by the participants during the test was greater under the fast/loud condition. The results of that study demonstrated that music with a fast tempo and high volume could improve running performance. Similarly, the study by Becker et al. also showed that music before exercise provided an advantage in stimulating or distracting mental sensations from the exercise itself, leading to improved performance (11). Distracting mental sensations from the exercise itself was the explanation also provided by Patania et al. when discussing their findings (9). The effect of music on effort perception is implicitly confirmed in the study by Delleli et al., who found that combining a low dose of caffeine—a stimulating substance—with music during a warm-up is an effective strategy to improve exercise performance (12). Ballmann et al. examined the effects of listening to preferred and non-preferred music on a repeated sprint through the Wingate Anaerobic Test (WANT), showing that exercise motivation was higher and effort perception was lower under the preferred music condition (13). In addition, a study by Atan aimed to evaluate the effect of listening to music and its tempo during the Running-Based Anaerobic Sprint Test (RAST)—similar to WANT—under three conditions: slow rhythm music, fast rhythm music, and no music (14). In contrast to other studies, no significant differences were found between the three conditions regarding power, heart rate, and blood lactate concentration, concluding that listening to music could not improve anaerobic performance (14). It appears that preferred music has a greater ability to divert attention away from the task toward external musical stimuli. This greater attention to music during exercise results in a lower perception of effort through four mechanisms: motivation, dissociation [quantified as lower rating of perceived exertion (RPE)], acute recovery, and affective responses (13). Music responses are subjective and affect many factors, including gender, age, and intrinsic and extrinsic motivation. Therefore, it is difficult to compare performance when listening to music between individuals due to their individual musical tastes (preferred vs. non-preferred music types). In fact, Nakamura et al. observed that non-preferred music worsens the dissociative mechanism by improving the rating of perceived exertion during physical exercise (15). Although evidence shows that listening to music is more effective in resistance training and endurance activities, the beneficial mechanisms that music promotes are not yet fully understood and clarified.

The present study aimed to investigate and confirm the efficacy of listening to preferred music during repeated high-intensity efforts on motivational level, perceived exertion, and biomechanical performance in sub-élite athletes. A better understanding of this topic may help to improve athletes’ response to training protocols or patients’ compliance and adherence to rehabilitation treatments based on anaerobic exercise. In particular, the present study investigated the mechanical output in terms of power, perceived effort in terms of RPE, and psychological response in terms of a visual analog scale (VAS), related to RAST administration performed with and without music.

## 2 Methods

### 2.1 Design setting

This study involved a repeated measures within-group design to investigate the effects of listening to music during anaerobic performances under two separate conditions: with preferred motivational music (≥120 bpm) or without music. The eligibility inclusion criteria were as follows: (a) no history of musculoskeletal injury in the lower limbs within the past 3 months; (b) weekly training routine of at least 3 days/week, 90 min per session; and (c) being affiliated with a sport federation. The exclusion criteria were as follows: (a) the presence of cardiopulmonary illness, (b) inadequate training volume in the previous week (<2 training sessions per week), (c) history of febrile illness in the last 2 weeks, (d) consumption of medications for at least 3 months before the study, and (e) consumption of caffeine or alcohol prohibited on the day before the study, as well in the hours immediately before or during the experimental protocol. All participants were informed of the purpose and risks of the investigation and provided signed written informed consent for participation. The study was approved by the Ethical Committee of the University of Insubria (protocol no. 0035460) in accordance with the declaration of Helsinki.

### 2.2 Participants

The sample size calculation was established via a power analysis [α = 0.05, β = 0.95, effect size (ES) = 0.7] based on a previous study examining the primary outcome [peak power output (PPO) during RAST test] (14) and establishing a sample numerosity of 25 individuals who would be reached using G*Power software. After the enrollment stage, 28 participants were screened. Six individuals were not considered because of the exclusion criteria and four individuals dropped out during the experimental procedures because of a worsening of their clinical conditions or family commitments. Thus, 18 individuals [men, *n* = 12; women, *n* = 6; mean age 22.2 ± 2.1 years; mean height 175.3 ± 8.0 cm; mean weight 66.4 ± 10.6 kg; mean body mass index (BMI) 21.5 ± 2.2 kg/m^2^] were included in the final sample size. All participants were voluntarily recruited in the Varese area, Italy. Weight was measured to the nearest 0.1 kg with a scale [model 770; SECA, Hanover, MD, USA (15)]. All athletes performed at a sub-élite level, engaging in competitive or non-competitive track-and-field and football disciplines (16). We chose a culturally uniform sample with similar training experience to reduce the influence of varying cultural backgrounds on participants’ psychophysical responses to music (17). Before taking part in the testing procedures, all participants were asked to abstain from vigorous activity in the 24 h preceding the test and to refrain from nicotine and alcohol in the 12 h preceding the test (18).

### 2.3 Procedures

**Figure 1:**
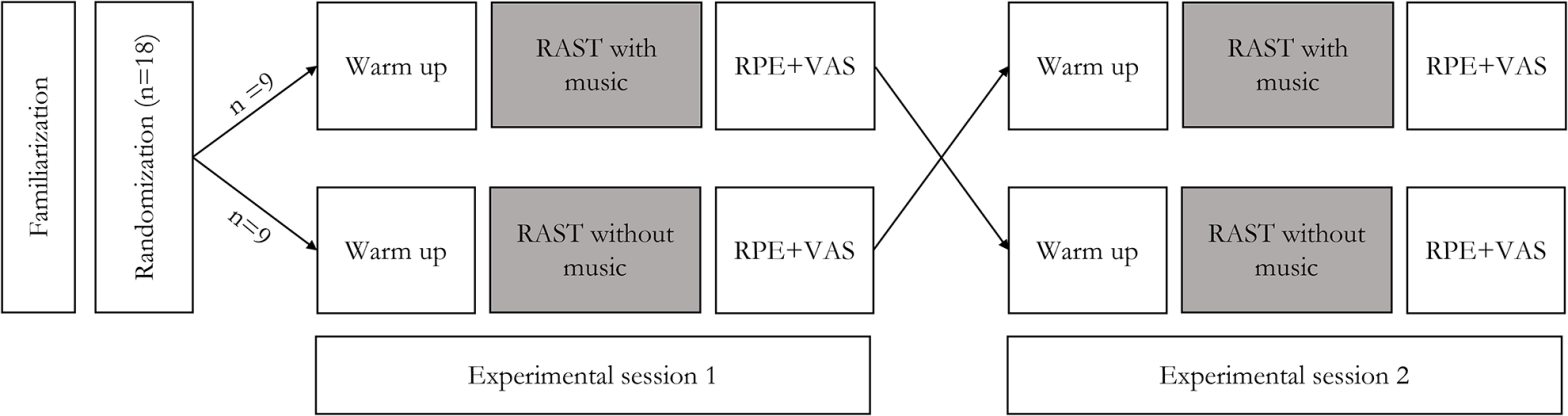
Experimental design setting. RAST, running-based anaerobic sprint test; RPE, rating of perceived exertion; VAS, visual analog scale.

Anaerobic performance was tested using RAST, a test developed by Wolverhampton University (UK) to assess anaerobic power (19, 20). Compared to the WANT test, RAST allows individuals to perform more sport-specific movement patterns using running as the main form of locomotion. Each participant was tested on two conditions (i.e., “with music” and “without music”) on the same track-and-field training center and at the same time of the day to avoid possible variations. Participants were tested in two experimental sessions 1 week apart: 1 day under the “with music” condition and 1 day under the “without music” condition. Testing protocols were provided in a random order to reduce the order effect of any conditions or minimize the risk of bias during the trial (21). Nine participants (six men and three women) performed the “with music” condition in experimental session 1 and the “without music” condition in experimental session 2, whereas this order was inverted for the other nine participants (six men and three women). The experimental design is illustrated in Figure 1.

Before the “with music” condition, athletes were asked to create a customized playlist on Spotify (Spotify Technology SA, Stockholm, Sweden) of five songs they preferred from any musical genre. All songs respected a beat per minute range of 120–130 bpm measured via the Tunebat website (https://tunebat.com). In fact, previous studies observed that music above this threshold can be stimulating and motivating, thereby improving sports performance (6, 11, 15). As stated above, the existing literature shows that in the preferred music condition, exercise motivation is significantly higher and RPE is significantly lower (6, 13, 15, 18). Since musical preference is an important factor in determining the ergogenic potency of music, it was more appropriate to have the participants choose the music.

In the 2 weeks before testing, all participants were informed about and familiarized with the procedures. All athletes were to listen to music through their headphones that were connected to their mobile phones. The volume was controlled by each participant before the test and set at a constant, medium level throughout the duration of the test (22) to respect adequate subjective comfort. Each participant had to be able to clearly hear the voice of someone nearby, despite the music playing through his/her headphones.

In addition, under the “without music” condition, all participants still wore their headphones but switched them off so as not to cause any potential bias effect. After a standardized 8-min warm-up (i.e., including 4 min of low-intensity running and 4 min of low-intensity jumps), the preferred music was turned on during the passive recovery of 3–5 min. Subsequently, the athlete had to complete six 35-m sprints at his/her maximum velocity while respecting 10 s of interval rest between each bout and continuously listening to the preferred music. The next sprint started from the opposite end of the first measurement in the same way. Specifically, under the “with music” and “without music” conditions, the participant received both the vocal command “go” immediately after the countdown and the visual signal consisting of the downward movement of the tester upper limb. This protocol was repeated at the test's beginning and the end of the 10-s recovery period between sprints. Finally, as for the “without music” condition, the same procedures were observed without listening to music through headphones.

### 2.4 Data collection

Once the athletes reached the test site, they were asked to record the following information: their age (years), height (cm), and body mass (kg) and to then determine their BMI. Performance times were recorded using Witty photocells (Microgate Srl, Bolzano, Italy) (23). At the end of each trial, participants were asked to measure their perceived level of exertion during the test, using the Borg RPE (24), a scale in the range of 6–20 (from no exertion to maximum exertion), and completing a VAS to assess subjective enjoyment of music on a 10 cm horizontal line, where the extremes indicate “minimum enjoyment” at point zero and maximum enjoyment at point 10 (25). No visual feedback or verbal encouragement was provided during the test. The testing sessions were conducted by AB, a Master of Science student in the Preventive and Adapted Physical Activity Degree Course with extensive track-and-field experience (26).

After completing RAST under both conditions, the PPO relative to the times of each sprint was calculated in watts using the formula in Equation (1):

(1) $$\mathrm{PPO}=\frac{\mathrm{body}\;\mathrm{mass}\times \mathrm{distanc}e^{2}}{\mathrm{tim}e^{3}}$$

From the power values of the six sprints, the following variables were determined: maximum power (Pmax), which provides information on the strength and speed of the test and represents the maximum PPO among the six sprints; mean power (Pmean), which provides information on the athlete's ability to maintain power over time and represents the average PPO over the six sprints; and minimum power (Pmin), i.e., the minimum PPO among the six sprints (19).

### 2.5 Statistical analysis

Data are presented as box-and-whisker plots and mean ± standard deviation. Preliminary to the comparison between conditions by paired statistical tests, the normality of the distributions of the difference between observations with and without music, Δ_i_, was verified using the Shapiro–Wilk normality test. If the hypothesis of normality was not rejected, the two conditions were compared using the paired Student's *t*-test or the Wilcoxon signed-rank test.

The ES was quantified by Cohen's *d* index for paired data. Cohen's *d* statistics quantify the absolute value of the difference between the means of two samples in relation to the dispersion of the samples quantified as standard deviation (25). For paired observations, the mean of the differences (*M*) is scaled by the standard deviation of the differences (SD) as in Equation (2):

(2) $$d=\frac{\left|M(\Delta_{i})\right|}{\mathrm{SD}(\Delta_{i})}$$

According to the original classification (27) and its successive expansion (28), magnitudes of *d* <0.2 indicate a very small ES, between ≥0.2 and <0.5 a small ES, between ≥0.5 and <0.8 a medium ES, between ≥0.8 and <1.2 a large ES, and ≥1.2 a very large ES (29). A significance level of 0.05 was used; therefore, a *p*-value <0.05 was considered statistically significant.

## 3 Results

**Figure 2:**
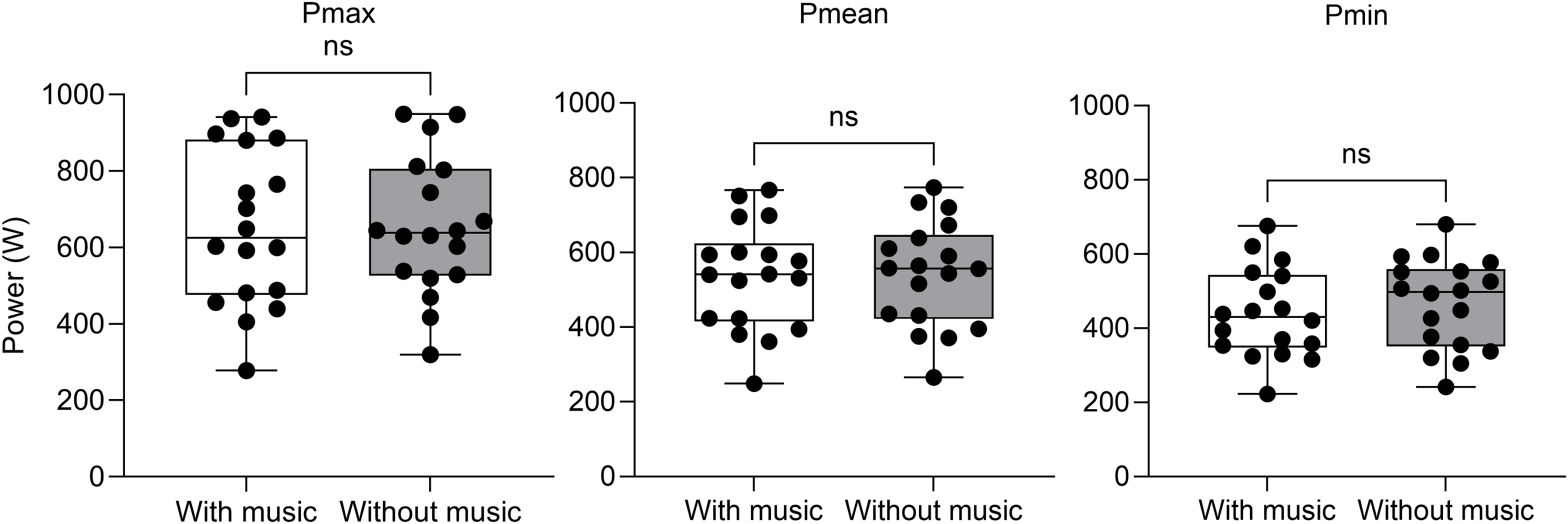
Box-and-whisker plots of the maximum (Pmax), mean (Pmean), and minimum (Pmin) peak power output over the six sprints of the RAST test separately under conditions with and without music. Boxes indicate the first, second, and third quartiles, whiskers represent the extreme values, and dots represent individual values. ns, not significant.

Variables that did not pass the normality test were Pmax, Pmin, and RPE. The participants’ overall results are summarized in Table 1. The values obtained under the musical conditions revealed a mean power output characterized by Pmax of 652.5 ± 203 W, Pmean of 536.0 ± 142 W, and Pmin of 439.1 ± 120 W. Conversely, the non-musical condition showed the following mean values: Pmax 654.6 ± 179 W; Pmean 541.9 ± 140 W; and Pmin 466.6 ± 121 W. Figure 2 shows Pmax, Pmean, and Pmin under the two conditions. No significant differences were observed between listening and not listening to music for Pmax (*p* = 0.500; ES = 0.04, very small), Pmean (*p* = 0.250; ES = 0.28, small), and Pmin (*p* = 0.053; ES = 0.52, medium).

**Table 1:** Measured separate outcomes in the “with music” and “no music” conditions (mean ± standard deviation).

Condition	Pmax (W)	Pmean (W)	Pmin (W)	RPE (a.u.)	Motivation VAS (cm)
With music	652.5 ± 203	536.0 ± 142	439.1 ± 120	15.2 ± 1.6	7.9 ± 0.9
Without music	654.6 ± 179	541.9 ± 140	466.6 ± 121	15.4 ± 1.7	6.4 ± 1.1

RPE, rating of perceived exertion; VAS, visual analog scale.

Pmax, Pmean, and Pmin are maximum, mean, and minimum peak power output in Watts.

**Figure 3:**
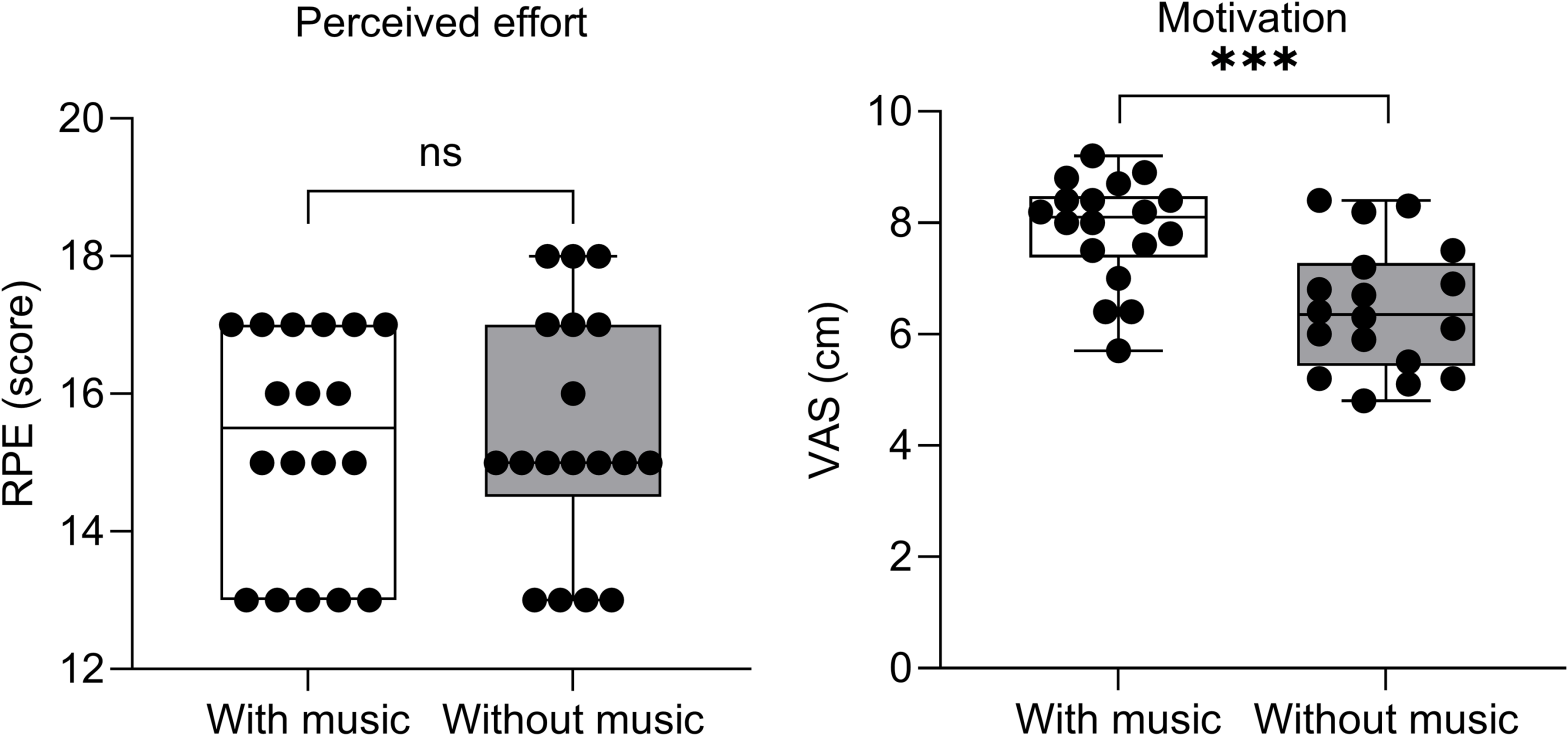
Box-and-whisker plots of perceived effort (as RPE) and motivational level (as VAS) post-test with and without music. Boxes indicate the first, second, and third quartiles, whiskers represent the extreme values, and dots represent individual values. ***Differences between conditions significant at *p* < 0.001. ns, not significant.

Figure 3 shows RPE and VAS. The difference between conditions was not statistically significant for RPE (with music 15.2 ± 1.6 a.u., without music 15.4 ± 1.7 a.u.; *p* = 0.710; ES = 0.08, very small), whereas VAS was significantly lower under the “with music” condition than the “without music” condition (with music 7.9 ± 0.9 cm, without music 6.4 ± 1.1 cm; *p* < 0.001; ES = 1.31, very large).

## 4 Discussion

The results of prior evidence indicated that an anaerobic exercise performed with music led to a significantly higher level of motivation than anaerobic exercise performed without music (5, 6, 8, 18, 30). Thus, this study aimed to examine and confirm the beneficial effect of music on maximal anaerobic performance in terms of mechanical output, namely power, metabolic input, as perceived exertion, and psychological response by motivation in sub-élite athletes. In this context, Ballmann et al. examined, using the WANT (similar to the RAST currently in use), the effects of preferred and non-preferred music on 14 male individuals showing that motivation was significantly higher under the preferred music condition (18). This could indicate that music during an anaerobic test can stimulate psychological and physiological functioning. On one hand, rhythmic elements enhance biomechanical efficiency by reducing the energy cost for a given workload; on the other hand, they promote higher levels of self-determined motivation, adding further benefits (2). Therefore, increases in motivation levels given by music, even if without affecting anaerobic performance, may play an important role in sustaining repeated maximal-intensity sprints. In our study, there were no significant differences between Pmax, Pmean, and Pmin under the two conditions. This result coincides with a previous study where, after RAST and WANT, no significant differences in Pmean values were found between the slow music condition, the fast music condition, and the condition without music (14). However, the lack of significant differences between conditions may, at least in part, be explained by variability in individuals’ prior training habits (17).

One of the main factors through which music imparts ergogenic benefits relates to the lowering of perceived exertion due to the increased attention given to music—i.e., an external focus—during exercise (13). Despite the lack of significance between the two conditions, in the present study, it appears that the musical condition resulted in slightly lower RPE values (Figure 3), compared with, for example, the study by Ballmann et al., who found a significant reduction in perceived exertion under the musical condition with respect to the non-musical condition (13). Some studies, in particular, examined participants during sub-maximal jogging and observed that music was more effective during low and moderate exercise intensities or in untrained participants (9, 30). Importantly, music can also be listened to after effort. In the study by Savitha et al., it was observed that relaxation with slow music after a period of moderate exercise provided a quicker recovery than fast music (31). It is worth noticing that our study protocol involved listening to preferred music (≥120 bpm) and not slow music. This could be a speculative reason why participants were unable to recover more quickly between sprints during the recovery period before the subsequent sprint. In addition, the study participants, coming from different training and sports backgrounds, may have had different levels of lactate tolerance and, therefore, become fatigued differently. Indeed, previous evidence has suggested that listening to music during anaerobic sprinting may affect long-distance runners and sprinters differently (32).

The present study has some limitations. A slow music condition was not included; songs and volume were chosen by each participant, adapted to his or her preference, and not in a standardized manner. Furthermore, the relatively low number of female participants compared to male participants prevented a comparison of the effects of preferred music on gender differences. Interestingly, some studies observed that the benefits of preferred music during exercise can be sex-dependent (7, 31) and that women exhibit greater emotional sensitivity to musical stimuli than men (29).

Finally, this study was not designed to examine the optimal musical intensity or the physiological response of preferred music on maximal anaerobic performance (6, 7). Monitoring physiological variables (e.g., heart rate and blood lactate) should be considered in future studies to help explain these mechanisms of preferred music on anaerobic performance (5, 9, 12, 33, 34). In fact, we did not investigate the effects of any musical tempo on heart rate or gait synchronization. This last effect was indeed demonstrated in a previous study (35). The above-mentioned effects of music are indeed worthy of future investigations.

Although the data collected in this study are preliminary, the results indicate that music has a positive effect on perceptual variables such as motivation and perceived exertion, even if the latter effect only came close to being statistically significant. In contrast, the present study noted that, during maximal anaerobic exercise, the music chosen could lead to a lower intensity of power, likely due to the difference in the tempo of the music but also to the gender differences between participants. The discrepancies between the different studies on the ergogenic effects of music highlight the need for further, more in-depth research to investigate the possible benefits of music during an anaerobic performance. Overall, integrating preferred music into an anaerobic performance could be an effective strategy, if not to optimize performance to at least improve general wellbeing and enjoyment. This may be of interest to increase training adherence, especially in individuals who are more prone to have a less active lifestyle (2).

## 5 Conclusion

Preferred music can increase motivation during high-intensity repeated exercise without improvements in anaerobic performance as confirmed in previous studies. Athletes can enhance their motivation by using music to strive for even higher goals in a natural and cost-effective way by improving their current performance level. However, although listening to music is currently prohibited in numerous competitive sports and events, this research could be applied to non-competitive scenarios. In fact, it is crucial to explore strategies to support adherence to high-intensity exercise beyond competition. Utilizing preferred music as a motivating effect can help alleviate the discomfort associated with unusual efforts when requested to perform exceeding usual limits. High-intensity exercise can enhance overall wellbeing in the general population and the incorporation of music during exercise may play a pivotal role in improving training adherence and motivation.

## Data Availability

The dataset supporting the findings of this study is available with access granted on justified request to researchers meeting the criteria for access to confidential data. Access requests should be directed to damiano.formenti@uninsubria.it.
